# Parent-Of-Origin Effects in Autism Identified through Genome-Wide Linkage Analysis of 16,000 SNPs

**DOI:** 10.1371/journal.pone.0012513

**Published:** 2010-09-02

**Authors:** Delphine Fradin, Keely Cheslack-Postava, Christine Ladd-Acosta, Craig Newschaffer, Aravinda Chakravarti, Dan E. Arking, Andrew Feinberg, M. Daniele Fallin

**Affiliations:** 1 Department of Epidemiology, Bloomberg School of Public Health, Johns Hopkins University, Baltimore, Maryland, United States of America; 2 Department of Medicine, Center for Epigenetics, Institute for Basic Biomedical Sciences, Johns Hopkins University School of Medicine, Baltimore, Maryland, United States of America; 3 Robert Wood Johnson Foundation Health & Society Scholars, Columbia University, New York, New York, United States of America; 4 Department of Epidemiology, Drexel University, Philadelphia, Pennsylvania, United States of America; 5 Center for Complex Disease Genomics, McKusick-Nathans Institute of Genetic Medicine, Johns Hopkins University, Baltimore, Maryland, United States of America; Universite de Montreal, Canada

## Abstract

**Background:**

Autism is a common heritable neurodevelopmental disorder with complex etiology. Several genome-wide linkage and association scans have been carried out to identify regions harboring genes related to autism or autism spectrum disorders, with mixed results. Given the overlap in autism features with genetic abnormalities known to be associated with imprinting, one possible reason for lack of consistency would be the influence of parent-of-origin effects that may mask the ability to detect linkage and association.

**Methods and Findings:**

We have performed a genome-wide linkage scan that accounts for potential parent-of-origin effects using 16,311 SNPs among families from the Autism Genetic Resource Exchange (AGRE) and the National Institute of Mental Health (NIMH) autism repository. We report parametric (GH, Genehunter) and allele-sharing linkage (Aspex) results using a broad spectrum disorder case definition. Paternal-origin genome-wide statistically significant linkage was observed on chromosomes 4 (LOD_GH_ = 3.79, empirical p<0.005 and LOD_Aspex_ = 2.96, p = 0.008), 15 (LOD_GH_ = 3.09, empirical p<0.005 and LOD_Aspex_ = 3.62, empirical p = 0.003) and 20 (LOD_GH_ = 3.36, empirical p<0.005 and LOD_Aspex_ = 3.38, empirical p = 0.006).

**Conclusions:**

These regions may harbor imprinted sites associated with the development of autism and offer fruitful domains for molecular investigation into the role of epigenetic mechanisms in autism.

## Introduction

Autism is a neurodevelopmental disorder that is manifested in early childhood and is characterized by impairments in reciprocal social interactions and language, and a restricted range of behaviors and interests. Autism is considered a spectrum disorder (ASD) with heterogeneity in symptom presentation. Inheritance clearly plays a major role in susceptibility to autism [1], [2], [3], [4], [5](OMIM %209850), yet efforts to identify susceptibility genes have been complicated by the apparent heterogeneous and complex etiology of this disorder. While some important genetic discoveries have been made (reviewed in [6]), much of the heritable variation in autism remains unexplained.

Epigenetic factors, which are often heritable, yet not part of the DNA sequence, are one element which may contribute to this etiologic complexity. Imprinting is an epigenetic modification that is parental origin specific, leading to preferential expression of a specific parental allele in somatic cells of the offspring [7]. Mechanisms such as DNA methylation, RNA-associated silencing and histone modification cause relative silencing of a specific parental allele. The vital role of imprinted genes in mammalian prenatal growth and development is shown most clearly by the abnormal development and early demise of embryos that inherit two copies of either a maternal or paternal genome, rather than the usual one of each [8]. In addition, the fact that many known imprinted genes are expressed in the brain (reviewed in [8]) suggests that such genes could play a role in autism, which is believed to have underpinnings in neuroanatomic differences that arise prenatally [9], [10]. The genetic disorders Prader-Willi and Angelman syndromes, which result from defects in imprinting or the loss of expression of imprinted genes in the chromosomal region 15q11-q13 [11] are associated with autistic features and diagnoses [12], [13], [14], [15], [16], and maternally transmitted abnormalities of chromosome 15 have been detected in autistic patients [12], [17], [18].

If imprinting plays a role in the heritable etiology of ASD, the power of linkage analyses to identify susceptibility loci may be improved by accounting for allelic parent-of-origin. This has been observed for specific autism-implicated genomic regions such as 7q, where both paternal and maternal allele sharing have been observed to account for the linkage to an autism locus in this region [19]–[20]. In a follow-up of previous linkage findings, Liu et al. reported partitioning of IBD sharing per parent on chromosomes 5, 16, 18 and 19, with both maternal and paternal peaks observed on chromosomes 5 and 19, suggesting the presence of multiple loci with parent-of-origin effects [21]. Arking et al. identified linkage and association with the *CNTNAP2* gene (contactin-associated protein-like 2) using genome-wide SNP analyses [22]. Further characterization of this signal showed maternal-specific parent-of-origin effects among heterozygotes.

To date, however, no genome-scale parent-of-origin-specific linkage analysis has been reported for ASD. Here we apply parent-of-origin linkage analysis to the genome-wide SNP data recently reported by Weiss et al. in a common set of multiplex autism families [23].

## Methods

### Subjects

The samples used here were previously described by Weiss et al. [23]. Nine hundred ninety three (993) families (896 affected sibling pairs) from the AGRE (Autism Genetic Resource Exchange) sample and 223 families (174 affected sibling pairs) from the NIMH (National Institute of Mental Health) Autism Genetics Initiative were included. AGRE families with a child diagnosed with an Autism Spectrum Disorder (ASD) based on evaluation by the Autism Diagnostic Interview-Revised (ADI-R) [24] were recruited from across the US. Further information on participant recruitment and study procedures has been described elsewhere [25] and is available on the program website (www.agre.org). From AGRE, we considered children with autism, “not quite autism (NQA),” or “broad spectrum” as affected family members to encompass those with related disorders such as Aspergers syndrome and PDD-NOS. Information on participant recruitment and study procedures for the NIMH sample is available on the program website (www.nimh.nih.gov). We selected NIMH families with a child diagnosed with an Autism Spectrum Disorder based on evaluation by the Autism Diagnostic Interview-Revised (ADI-R) and ADOS instruments. The combined data set, consisting of 1,216 nuclear families, was used for genetic analyses. All families used in our analyses had at least one genotyped parent; 89.4% had genotypes for both parents.

All samples used in this study arose from investigations approved by the appropriate Institutional Review Boards for institutions where participants were recruited, evaluated, or where genotype data were generated. Written informed consent was obtained for all adult study participants; for children under age 18, both the consent of the parents or guardians and the assent of the child were obtained. This secondary analysis of de-identified data was considered to be exempt from IRB review.

### Markers

SNP genotyping was previously described [23]. The AGRE samples were genotyped on Affymetrix 5.0 chips at the Genetic Analysis Platform of the Broad Institute, using standard protocols. The NIMH autism samples were genotyped at the Johns Hopkins Center for Complex Disease on the Affymetrix 500K (Nsp and Sty) and 5.0 platforms using similar standard protocols. We selected an extremely high quality set of SNPs for linkage analysis, including only SNPs genotyped in both data sets with 99.5% concordance and ≤1 Mendelian error. Linkage analysis involving high densities of markers, where clusters of markers are in linkage disequilibrium (LD), can lead to biased results [26]. To alleviate these concerns, we analyzed a pruned set of 16,311 highly polymorphic, high-quality autosomal SNPs that did not contain any two nearby markers correlated with r^2^ >0.1, providing a marker density of 0.25 cM. Genetic distances were taken from the Affymetrix Genetic Map (www.affymetrix.com/estore/browse/products.jsp?productId=131459&categoryId=35906#1_3)[26].

### Linkage analyses and simulations

Parametric and non-parametric parent-of-origin linkage methods were applied. Parametric linkage analysis was conducted using GENEHUNTER-IMPRINTING 2.1 (GHI) [27], with 1216 informative families. Using GHI, for each chromosome, we began with a fully penetrant maternal or paternal model with no phenocopies. Allele frequencies were estimated using the founders, and LOD scores under heterogeneity (HLOD) were calculated at five equally spaced intervals between markers. For each suggestive linkage result (HLOD≥2 for either maternal or paternal models), additional models were fit allowing for reduced penetrance or increased phenocopy rates to assess the sensitivity of the linkage signal to alternative parameters using the following procedure. We began by reducing the parent-specific penetrance at increments of 0.2 (*i.e.*, penetrances of 1, 0.8, 0.6, 0.4, 0.2) and by increasing phenocopy rates at similar increments. Empirical p values for these sensitivity results were estimated similarly to the initial genome-wide empirical p values (see below), but with the optimized parametric model applied for all locations.

For non-parametric linkage analysis, maximum likelihood estimates of allele sharing at each locus were computed using the ASPEX “sib_ibd” command. The “sex_split” option was implemented to evaluate evidence for linkage based on maternal and paternal sharing separately. 1070 affected sibling pairs were informative (had ≥1 parental genotype) for these analyses. LOD scores indicate the log ratio of the likelihood of the marker data at this position with the MLE estimate of the sibling recurrence risk ratio, versus the likelihood assuming a sibling risk ratio of 1.

Empirical genome-wide p values were calculated via simulation. The program Merlin [28], [29] was used to generate replicates of families identical to those in our sample with respect to marker informativeness, spacing and missing data patterns, and with affection status preserved but no relationship between simulated genotypes and affection. Merlin assigned random genomes to founders according to allele frequencies at each marker, then passed chromosomes through the pedigree using the relationships specified in the original pedigree file and recombination fractions specified by our genetic map. Linkage analyses were then performed on these unlinked replicates and genome-wide empirical p values were estimated by extrapolating results for chromosome 1 to the whole-genome level, assuming chromosome 1 represents 0.1 of the genome. Empirical genome-wide p values reported here were based on 2,000 replicates.

## Results and Discussion

**Figure 1:**
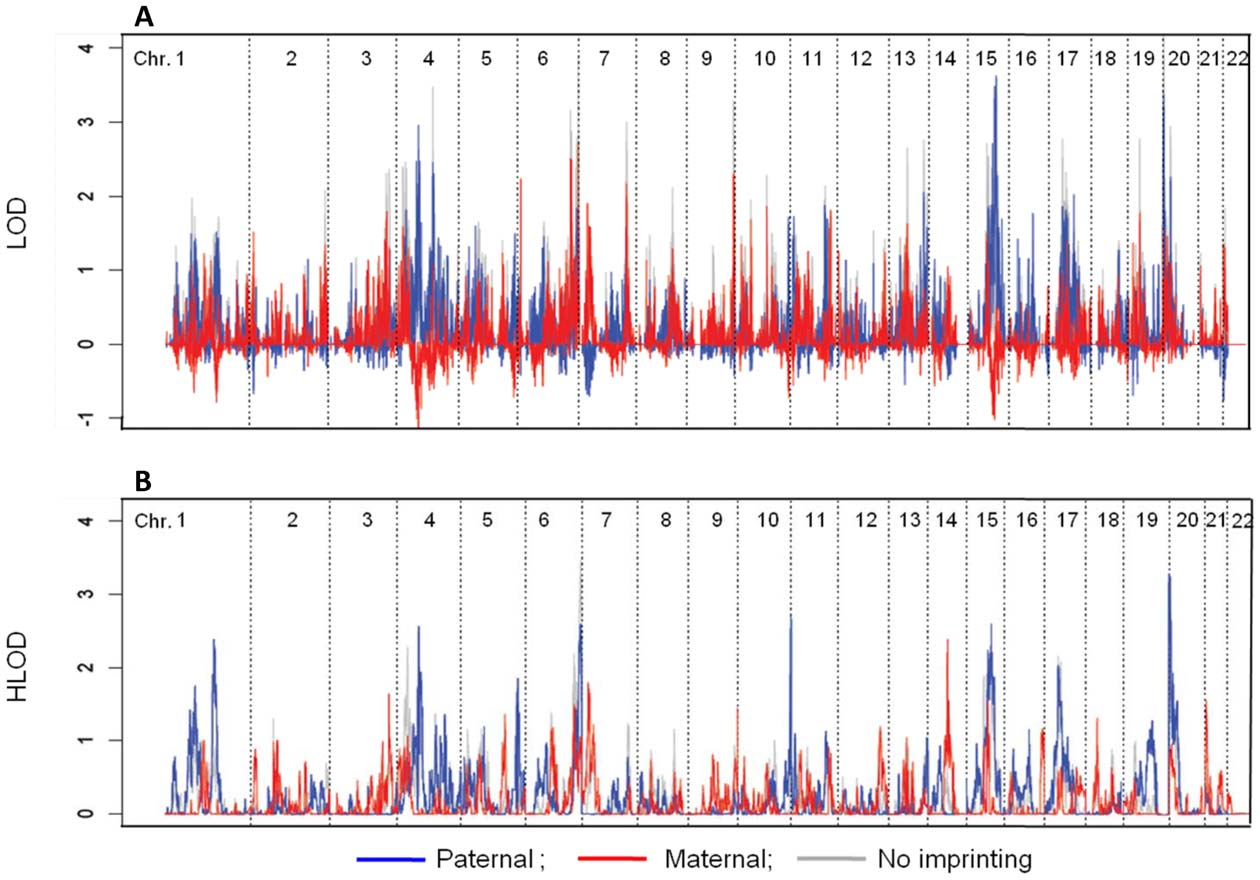
Results of Genome-wide Linkage Analysis. Panel A: Parametric results. Panel B: Allele sharing results. Maternal scores are shown in red; paternal scores are in blue; no-imprinting scores are shown in gray.

**Figure 2:**
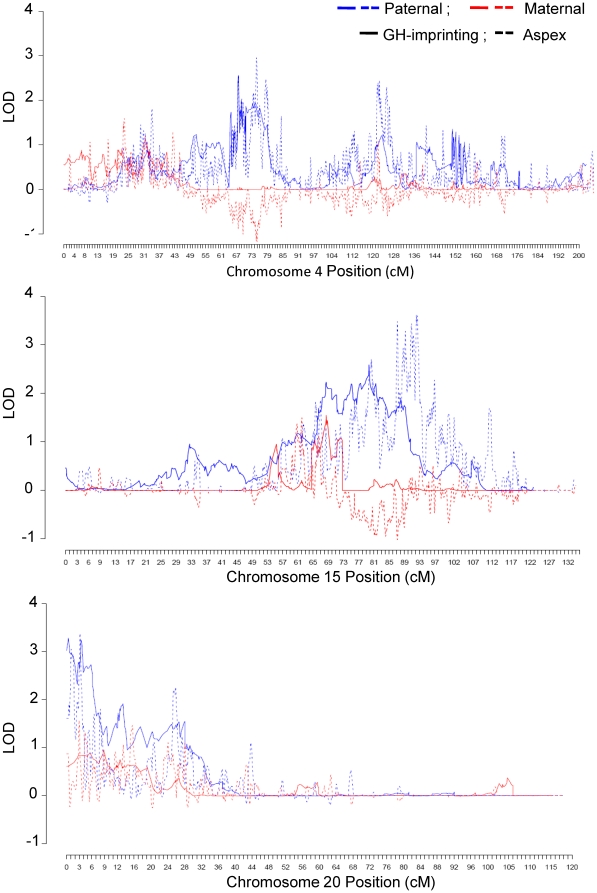
Multipoint Parametric and Allele-sharing LOD scores on Chromosomes 4, 15 and 20. Maternal scores are shown in red; paternal scores are in blue.

Genome-wide results for maternal and paternal linkage analyses via both parametric and non-parametric methods are shown in Figure 1. The highest HLOD and LOD signals on each chromosome are shown in Table 1. The final models for the strongest linkage signals from sensitivity analyses of parametric models are shown in Table 2. Paternal peaks based on both parametric and allele-sharing analyses were observed on chromosomes 4 (rs6826933:rs17088473, HLOD = 3.79, p<0.005; LOD = 2.96, p = 0.008; Table 1, Figures 1, 2), 15 (rs11855650:rs10520676, HLOD = 3.09, p<0.005; LOD = 3.62, p = 0.003; Table 1, Figures 1, 2) and 20 (rs16999397:rs200888, HLOD = 3.36, p<0.005; LOD = 3.38, p = 0.006; Table 1, Figures 1, 2). All p values reported reflect genome-wide testing based on simulation. Additional paternal peaks with HLODs >2 were observed on chromosomes 1, 6, 10, and 17 in the parametric analyses, with empirical genome-wide p values ≤0.01 (Table 1).

**Table 1:** Highest Linkage Peaks Per Chromosome.

	Parametric (GHI)					Allele-Sharing (Aspex)				
Chr	Map (cM)	No-imp HLOD	Pat. HLOD	Mat. HLOD	Pval**	Map (cM)	No-imp LOD	Pat. LOD	Mat. LOD	Pval**
1	156.55	0.66	**2.38**	0	0.01	168.67	0.76	1.52	−0.76	0.17
	*164.99*		***2.54 (1A)*** *		*0.01*					
2	82.90	0.43	0.12	1.00	1.00	15.77	0.85	−0.66	1.52	0.21
3	189.11	1.03	0	1.64	0.22	192.74	1.67	−0.1	1.79	0.13
4	67.85		**2.55**		0.01	74.81	2.01	2.96	−0.95	0.008
	*67.85*	*1.99*	***3.79 (4A)***	*0*	*<0.005*	*123.65*	*3.13*	*2.45*	*0.68*	*0.02*
5	182.85	0.49	1.85	0	0.08	205.55	1.82	−0.42	2.24	0.05
6	177.24	3.46	**2.59**	1.01	0.01	188.29	3.87	1.15	2.72	0.02
	*175.93*		***2.72 (6A)***		*0.01*					
7	20.32	0.55	0	1.79	0.13	156.43	2.99	0.84	2.16	0.06
8	NA	<1	<1	<1	NA	120.57	1.952	0.63	1.29	0.31
9	158.19	0.73	0	1.44	0.76	156.77	2.00	−0.31	2.32	0.04
10	169.29	0.72	2.69	0	0.01	103.52	2.22	0.36	1.86	0.12
11	111.01	0.77	1.13	0	1.00	114.49	1.40	1.87	−0.48	0.08
12	134.99	0.30	0	1.19	1.00	29.46	0.80	1.14	−0.34	0.23
13	56.98	0.86	0.02	1.04	1.00	113.79	2.75	2.05	0.71	0.06
14	62.33	0.43	0	2.38	0.01	18.67	0.58	1.15	−0.56	0.37
15	79.68	1.78	**2.59**	0.03	0.01	92.16	3.52	3.62	−0.11	0.003
	*64.61*		***3.09 (15A)***		*<0.005*					
16	121.30	0	1.16	0	1.00	78.81	1.41	1.77	−0.35	0.10
17	45.23	2.15	2.03	0.58	0.01	81.45	1.56	2.02	−0.46	0.07
18	NA	<1	<1	<1	NA	91.97	1.33	1.27	0.06	0.3
19	92.91	0.16	1.27	0	0.92	37.73	2.75	0.99	1.77	0.13
20	3.38	3.04	**3.24**	0.83	<0.005	3.20	4.13	3.38	0.75	0.006
	*0.40*		***3.36 (20A)***		*<0.005*					
21	3.58	0.66	0.28	1.55	0.58	6.12	1.23	0.17	1.06	0.45
22	NA	<1	<1	<1	NA	2.25	0.60	−0.75	1.35	0.28

*Optimal parametric model for this linkage signal (see Table 2).

**Empirical genome-wide p values based on 5000 simulations.

**Table 2:** Optimized Parametric Models.

	Model	P(+/+)*	P(d/+)	P(+/d)	P(d/d)
***Primary models***	***paternal***	0.001	0.999	0.001	0.999
	***maternal***	0.001	0.001	0.999	0.999
***Optimal models*** **	***1A***	0.001	0.499	0.001	0.499
	***4A***	0.001	0.399	0.099	0.499
	***6A***	0.001	0.599	0.199	0.799
	***15A***	0.001	0.499	0.001	0.499
	***20A***	0.001	0.799	0.199	0.999

*Paternally inherited allele named first. +: wild-type allele; d: disease allele; P: penetrance.

**Parameter values for best linkage signal after exploratory sensitivity analysis.

Although significant maternal peaks were observed in both parametric and allele-sharing methods, no consistency in signal was seen. A significant maternal peak was observed on chromosome 14 in parametric analyses (rs923485:rs17177789, HLOD  = 2.38, p = 0.01), although this was not observed in allele-sharing analysis. Maternal allele sharing peaks were observed on chromosomes 5, 6, 7, and 9 (Table 1), although these were not seen in parametric models.

Given the vital role of imprinted genes in development, the fact that many known imprinted genes are expressed in the brain, and evidence of overlapping features in autism and imprinting disorders, we investigated the effect of incorporating allelic parent-of-origin into an autosomal linkage scan for autism. To our knowledge, this is the most extensive linkage analysis for parent-of-origin effects in autism to date. We found the strongest evidence for parent-of-origin effects on chromosomes 4, 20 and 15, implicating sites where imprinted loci related to autism may reside.

The section of chromosome 4 located between markers rs6826933 and rs17088473 showed several significant results in our analysis and spans the region between 4q12-4q13.2. Recently, Weiss et al. found an association between one SNP (rs17088254, p = 8.5×10^−6^) located on this region and autism using the same data without regard to parental origin. The strongest candidate gene in this region is *CLOCK*, which codes a protein regulating circadian rhythm and whose involvement in ASD was first proposed by Wimpory et al. [30]. The most consistent results reporting abnormal circadian rhythms in ASD concern the melatonin synthesis pathway. At least five independent groups detected abnormal melatonin levels in ASD [31], [32], [33], [34]. Several lines of evidence suggest that melatonin could modulate neuronal networks by influencing both the strength and the circadian oscillation of neuronal transmission [35], [36].

Analyses of a panel of microsatellite markers in 348 AGRE families from previously reported linkage analysis[21], [37] also showed paternal allele sharing on chromosome 4 (Figure S1). However, the peak using the microsatellite panel was 29cM away from the SNP peak (D4S1591:GATA30B11, LOD_pat_ = 2.96, p = 0.008). The location of this microsatellite peak also showed linkage in the SNP data, but it was not the highest SNP linkage peak on chromosome 4.

A region of chromosome 15 (15q23-15q25.3) also shows paternal linkage. This region was previously implicated using traditional linkage analysis in these SNP data [23], though it this was not the strongest linkage signal in that analysis. A genome-wide assessment of structural abnormalities in 427 unrelated ASD cases found a microdeletion of 4,289,500bp on 15q23-q24.2 associated with ASD [38]. This region includes the *RASGRF1* gene, a homologue of the imprinted *rasgrf1* in mouse [8]. The protein encoded by this gene is a guanine nucleotide exchange factor (GEF). Functional analysis has demonstrated that this protein stimulates the dissociation of GDP from RAS protein. Studies of the similar gene in mice suggested that the Ras-GEF activity of this protein in the brain can be activated by Ca2+ influx, muscarinic receptors, and G protein beta-gamma subunit. Mouse studies also indicated that the Ras-GEF signaling pathway mediated by this protein may be important for long-term memory. Others genes in this region with plausible connections to autism risk include *NRG4* (neuregulin 4) and *CHRNA3/B4* (cholinergic receptor, nicotinic). Genes in the neuregulin[39] and cholinergic families[40] have already been implicated in autism risk. The 15q23-q25.3 region also encompasses the *MTHFS* (5,10-methenyltetrahydrofolate synthetase) gene which is implicated in DNA methylation cycle and may be particularly important in an epigenetic mechanism of autism risk.

We have also reported a strong paternal linkage on chromosome 20p, which was previously implicated in the linkage analyses reported by Weiss et al[23]. In analyses of these data without consideration of parent-of-origin, this region achieved a LOD score in excess of 2.0. According our results using the same data set, this linkage is supported by paternal transmission. Deletions of the 20pter region have been reported in two distinct autism cases [41], [42]. The first patient presented an interstitial deletion in 20p11.22-p11.23 whereas the second, a 3-year-old boy with a moderate to severe mental retardation and autistic behavior patterns, carried a deletion at 20pter-p12.2. Moreover, this linked region encompasses the *SNPH* (Syntaphiliyn) gene. SNPH interacts with the synaptic vesicle-associated protein synaptobrevin/VAMP and the plasma membrane-associated protein SNAP25 to form the SNARE complex, which is required for synaptic vesicle docking and fusion. Expression of this gene appears to be brain specific.

Other suggestive parent-specific linkage regions are located throughout the genome (on chromosomes 1, 5, 6, 7, 8, 9, 10, 13, 14, 17 and 21). The paternally linked region on chromosome 1 (1q23-1q24.2) was previously associated with autism. Wassink *et al* report a male child with autism having a maternal uniparental disomy (UPD) of chromosome 1 [43]. Moreover, in a previous genome wide linkage analysis, Bartlett *et al*. also found a linkage between the 1q23-1q24 region and autism using an AGRE sample [44].

A maternally linked region was observed on chromosome 5, with the peak at 5p13.1. Recent genome-wide association studies have reported risk loci for autism at 5p14.1 [45], [46]. The Imprinted Gene Database (www.geneimprint.com) lists four predicted imprinted genes on chromosome 5; however the regions do not directly overlap the location detected by our analysis.

The signal found on 6q25.3-6q27 region was previously linked to autism [23], [47], [48]. Recently, Glessner et al. found that *PARK2* gene located on 6q25.2-6q27 was significantly enriched for CNVs and observed in the ASD cases only [49]. They identified a deletion of about 3 kb in *PARK2* allele inherited from father. *PARK2* is an ubiquitin-protein ligase, mutations of which cause autosomal recessive juvenile Parkinson's disease [50]. Moreover, several autism cases with subtle interstitial deletions in the q24-q26 region of the long arm of chromosome 6 have been reported [51]. Some imprinted genes have also been described in this 6q region like the SLC22A (solute carrier family 22) genes.

Two other groups have previously reported parent-of-origin linkage with autism for closely located loci on chromosome 7; one was a paternal contribution in the region 7q31.33-7q34 [19], the other showed a paternally derived locus more proximally located on 7q22.1-7q22.2 and a maternally derived locus on 7q32.1-7q32.2 [20]. In our analysis, the strongest chromosome 7 signal was at 156.43 cM on nearby 7q35 under the maternal model. While not our strongest parent-of-origin signal, this provides further support for the presence of a maternally expressed locus in this region. Indeed, this region encompasses the *CNTNAP2* gene, a member of the neurexin superfamily, that is significantly associated with autism susceptibility [22], [52], [53], [54], and has shown maternal transmission of risk [22].

Few previous studies have considered parent-or-origin effects in autism. Those that have used previous-generation marker sets and much smaller samples than the results presented here. Two previous studies observed parent-or-origin linkage on chromosome 7, but with different regions and types of parental sharing [19], [20]. We did not reach genome-wide significant evidence for maternal or paternal transmission on chromosome 7, although a non-significant maternal linkage is observed in a region overlapping the Lamb et al report. The regions identified in our analysis were not covered in previous parent-of-origin analyses, to our knowledge, with the exception of chromosome 15, where the Lamb et al did not see paternal sharing, but examined only a small number of sibling pairs and with few microsatellite markers.

In an attempt to detect loci with possible parent-of-origin effects, we used multiple statistical approaches, rather than relying on a single strategy. Consistent evidence of linkage across multiple methods increases support for a true linkage. However, the appropriate interpretation of inconsistent results across parametric and non-parametric analyses is not entirely clear. These may be due to chance findings in one analysis, or they may be true linkage that only one method was sufficiently powered to detect. For example, the chromosome 1 peak was significant only in the parametric analysis, a method which is more powerful given that the parameters are correctly specified. While it is unrealistic to believe that we could actually have specified the “correct” parameters given the complex nature of autism, those selected may have been sufficiently close. Peaks on chromosomes 6 and 9 were significant in the non-parametric ASPEX analysis; however, the parametric GHI analysis did not find significant peaks on these chromosomes, which may be due to selection of “incorrect” parameters for the models run.

These analyses considered as affected all children with an ASD, as defined by the ADI-R and ADOS in the NIMH sample. However, in AGRE, we included those with autistic disorder, as well as those with “not quite autism” and “broad spectrum” to encompass Asperger's and PDD-NOS. This may have contributed to some heterogeneity or misclassification in our data, but was considered more appropriately inclusive and comparable to the NIMH ASD families than excluding a larger number of AGRE families with an ASD other than autistic disorder.

Our results suggest the usefulness of genome-wide analysis with evaluation of parent-of-origin effects, although future studies are necessary to determine if these results can be replicated. Given the potential role for imprinting and other epigenetic mechanisms in neuropsychiatric disorders such as autism [55], the regions identified are good candidates for assessment of functional variants and their relationship to epigenetic marks such as methylation status on paternal and maternal DNA. These results could provide completely novel insight into the biology and pathogenesis of a common neurodevelopmental disorder.

## Supporting Information

Figure S1 Parent-of-Origin Linkage Analysis for Microsatellite Markers in 384 AGRE families. A: Parametric results. Panel B: Allele sharing results. Maternal scores are shown in red; paternal scores are in blue.
(0.68 MB TIF)
