# Supplementary figures and images for: Parent-Of-Origin Effects in Autism Identified through Genome-Wide Linkage Analysis of 16,000 SNPs
Source: PLoS One. 2010 Sep 2;5(9):e12513. doi: 10.1371/journal.pone.0012513 (PMC2932694; doi:10.1371/journal.pone.0012513)

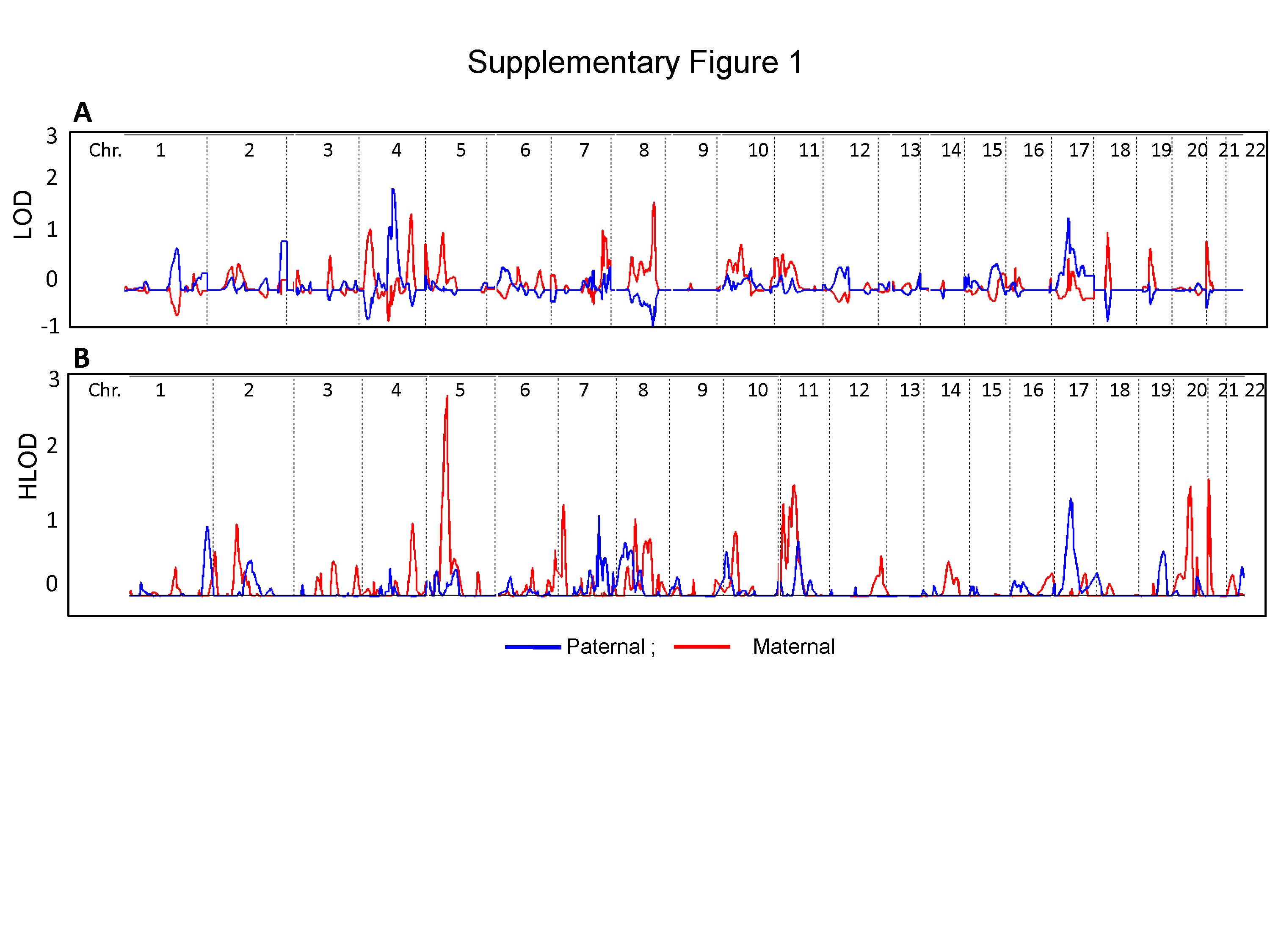

Supplement: Figure S1 — Parent-of-Origin Linkage Analysis for Microsatellite Markers in 384 AGRE families. A: Parametric results. Panel B: Allele sharing results. Maternal scores are shown in red; paternal scores are in blue. (0.68 MB TIF) [file pone.0012513.s001.tif]
